# Convective burn from use of hairdryer for heel warming prior to the heel prick test - a case report

**DOI:** 10.1186/1471-2431-11-30

**Published:** 2011-05-10

**Authors:** Robbie Ray, Yvette Godwin, Ashley Shepherd

**Affiliations:** 1 Speciality Training Registrar, Trauma and Orthopaedics, Trauma Unit, Royal Infirmary of Edinburgh, 16 Little France Crescent, EH16 4SA, Scotland, UK; 2 Consultant Plastic Surgeon, Department of Plastic Surgery, Royal Hospital for Sick Children, Sciennes Road, Edinburgh EH54 6PP, Scotland, UK; 3 Lecturer, School of Nursing, Midwifery and Health, University of Stirling, Stirling, FK9 4LA, Scotland, UK

## Abstract

**Background:**

Blood sampling through heel lancing is the most common invasive painful procedure performed on newborn infants.

**Case Presentation:**

We report the case of a five day old infant who sustained burns to the left foot and leg after the mother's hairdryer was used by the midwife to warm the baby's heel prior to capillary blood sampling (CBS) with an automated device.

**Conclusion:**

Heel warming is not recommended for routine CBS although it is often practiced. If pre-warming is to be practiced, standardised devices should be used rather than improvised techniques. This will reduce the risk of injury to these infants.

## Background

Capillary blood sampling (CBS) is routinely offered to all newborn infants born in the United Kingdom to identify babies who may have rare but serious conditions for example phenylketonuria, congenital hypothyroidism, or cystic fibrosis. CBS uses dried spots of blood obtained by heel prick and collected on filter paper. Current blood spot sampling guidelines suggest that additional warming of the foot is not required before heel puncture but that the heel should be warm [1]. Although the heel prick procedure is relatively easy to complete, a recent study has highlighted great variability in the technique among midwives [2]. Problems with CBS still exist including pain for the baby [3], anxiety for the parents [4] and complications from mild bruising [5]. The case reported below describes the injuries sustained by a five day old infant after heel heating.

## Case Presentation

A five day old baby presented with burns to the left foot and leg after having a heel prick test performed. A community midwife had visited the parents' home four hours earlier and to facilitate blood sampling, had used the mother's hairdryer to warm the baby's foot. The hairdryer was set on a high setting and was held about six inches from the baby's foot for less than one minute. The baby became distressed as soon as the heel prick was administered, and it was an hour after the procedure when the baby was finally consoled that the mother noticed erythema and blistering over the baby's foot and leg.

**Figure 1:**
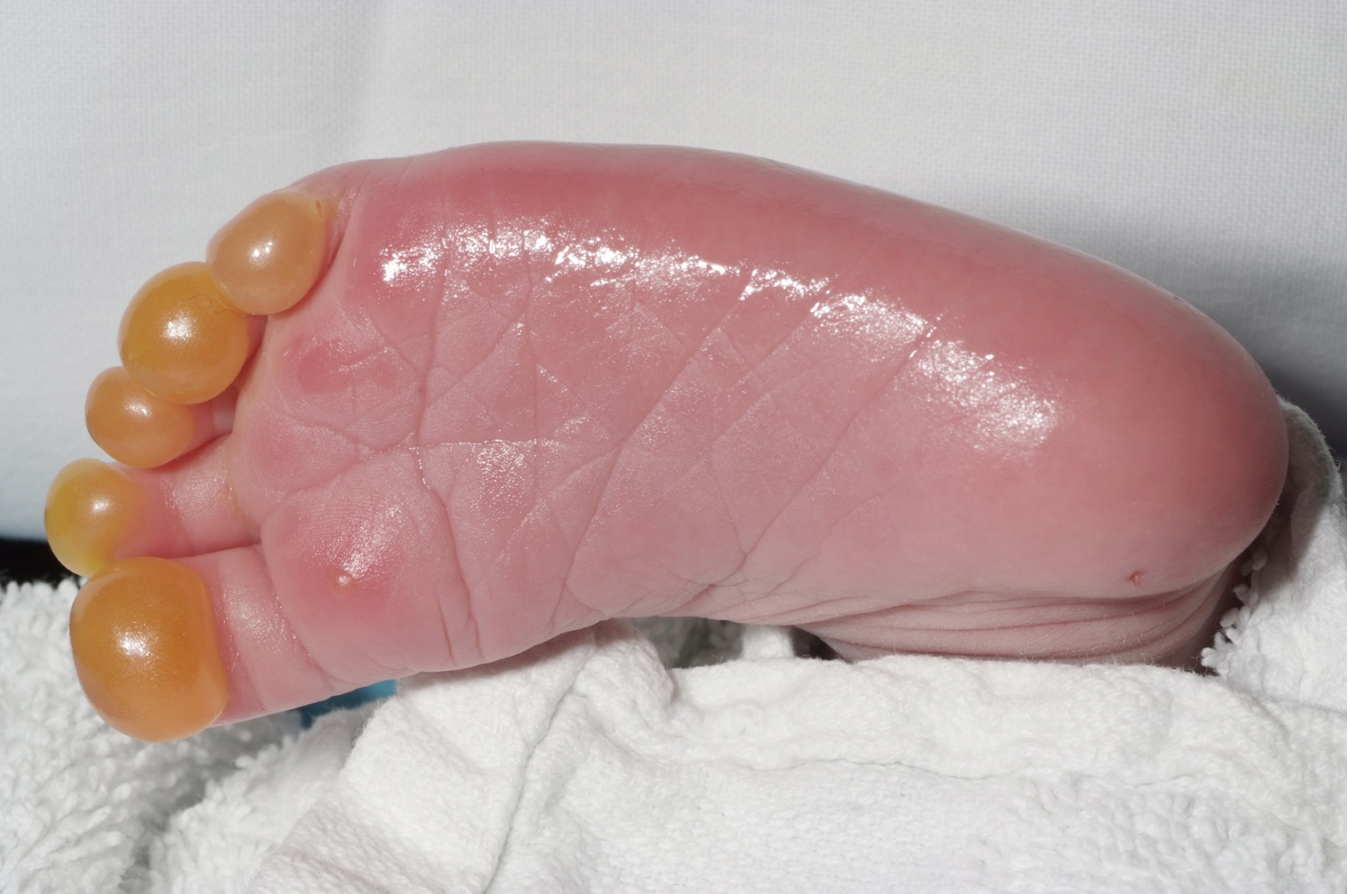
**Photograph taken on admission to hospital**.

On examination the baby had serous blistering over all the toes suggesting a superficial partial thickness burn (Figure 1). Swelling and erythema extended from the leg to the knee and the infant was clearly distressed and obviously in pain from the injury.

During the next 48 hours there was concern regarding the possible progression of the depth of the burn hence the infant was admitted for observation and wound dressing. The blisters were deroofed and conservative treatment with dressings was pursued. At three weeks post injury, there was complete closure of the burns wounds. A final review, in outpatient's clinic, showed minor maturing scars on the pulps of the 2^nd^-5^th ^toes. No long-term consequence of these minor scars, or scar contracture was anticipated and the patient was discharged.

Blood sampling through heel lancing is the most commonly performed invasive painful procedure in the newborn [6]. A literature review was performed using Medline and Cinahl databases for papers published between 1992 and 2011 with the search terms - heel prick, capillary blood sampling and warming in multiple combinations.

It is acknowledged that the heel prick procedure can be uncomfortable for the child [7] so it is imperative to perform the procedure as efficiently as possible with the minimum of trauma to the infant. Analgesia in the form of breast feeding, non-nutritive sucking and a dose of oral sucrose or glucose is recommended [8,9]. Automated devices which allow for a standard safe penetration of the vascular bed have been recommended for the heel prick procedure [10]. Warming of the heel prior to incision is based on the supposition that an increase in skin temperature causes an increase in blood flow which should provide a larger volume of blood to sample. However, evidence from videophometric microscopy analysis has shown that capillary blood flow is unaffected over the range of temperature that is increased by heel warming [11]. Furthermore, randomised control trials have shown that there is no increase in the volume of blood expressed or reduction in complications such as pain or bruising when the heel is warmed [12,13].

Prior to incision, warming of the heel can be performed if the foot is clearly cold using a specifically prepared gel filled heel warmer (Rapidaid [14]). These warmers are activated by manipulation of a trigger disc which heats the gel to 40°C via an exothermic reaction and then secured in place with tape. Alternatively the infant's heel can be heated using water warmed to 42°C which must be checked by the midwife prior to heating [10,12,13]. There are currently no reports in the literature of using hairdryers to warm the heel. The inquiry held after this specific case has resulted in national guidance being issued to midwives to avoid unknown heating sources for pre-warming of infants' feet.

One previous paper [15] and a report from the New Zealand health commission refer to burns caused by heel warming [16]. These injuries were sustained when a midwife used a nappy soaked in hot water and a cup of water boiled from a kettle respectively. This case study is the first report of injuries to be sustained from a hairdryer burn.

CBS is an important public health screening measure that allows health professionals to detect potentially harmful conditions and treat them at an early stage. For some conditions management can be initiated which will greatly reduce the deleterious effects and complications caused to the child. For example if started early, treatment for infants diagnosed with phenylketonuria is highly effective at preventing development of serious mental disability [17]. The UK newborn screening programme centre, funded by the Department of Health does not advocate routine heel warming in their most recent guidelines [1,17] and the literature does not support the need for heel warming before the heel prick test [12,13]. Intense heat or prolonged exposure to a heat source would have been required to cause a partial thickness burn in glaberous skin, as found on the sole of the foot in this case study infant. Therefore, if the heel is very cold and does have to be warmed, safe methods such as a standardised heel warmer should be used so that reliable temperatures can be reached every time and the baby is not at risk of burns [12]. However, further research is clearly needed to asses the usefulness of heel warming in these infants and the most effective way to do this.

## Conclusions

The heel prick procedure used by midwives today is similar to that followed when the heel prick test was first introduced despite research findings which contradict many of the steps [18]. The techniques used to obtain a sufficient sample are variable and one possible reason for this is that the procedure is taught by midwife mentors who tend to teach their own preferred method [2] rather than following the most recent research based guidelines [1]. Due to the problems discussed here and the new findings reported in this case study, perhaps the time has come for the heel prick test to be an accredited skill requiring a certificate of competence.

## Consent

Written consent was obtained from the infants parents for publication of this case report and the accompanying images.

## Competing interests

The authors declare that they have no competing interests.

## Authors' contributions

RR performed the initial literature review and first draft of the case report, liaised with the family and obtained consent. YG is consultant under whom this patient was treated and made contributions in drafting the final manuscript. AS has offered her expert knowledge in this area of care, made useful contribution in drafting this manuscript and in the review of the literature. All authors have read and approved the final version of this manuscript.

## Pre-publication history

The pre-publication history for this paper can be accessed here:

http://www.biomedcentral.com/1471-2431/11/30/prepub
